#  Para uma sociologia do câncer 

**DOI:** 10.1590/0102-311XPT161023

**Published:** 2023-12-04

**Authors:** Ana Beatriz Duarte Vieira, Gracielle Soares Silva Ruas, Nelson Filice de Barros

**Affiliations:** 1 Universidade de Brasília, Brasília, Brasil.; 2 Universidade Estadual de Campinas, Campinas, Brasil.

O livro *Survivorship: A Sociology of Cancer in Everyday Life* de Alex Broom & Katherine Kenny ^1^, publicado em 2021, enfatiza novos olhares para compreender os processos pelos quais passam as pessoas sobrevivendo ao câncer e os significados de ver, tratar e cuidar de pessoas na “guerra contra o câncer”. Seus sete capítulos resultam de pesquisas qualitativas realizadas durante o período de 2000 a 2020, na Austrália, com pessoas sobreviventes ao câncer, seus familiares, cuidadores e profissionais de saúde.

Na introdução, o leitor é apresentado à história centrada na doença e na indústria do tratamento da sobrevivência ao câncer nas últimas décadas. Entretanto, os capítulos subsequentes apontam que a sobrevivência ao câncer “*governa corpos, identidades e relações sociais*” (p. 8) e, de forma assimétrica, espelha as injustiças sociais, a invisibilidade das dimensões subjetivas, os fluxos temporais das relações, os entrecruzamentos institucionais e apelos discursivos.

O capítulo 1, *Bodily Becomings*, apresenta o câncer para além de um corpo adoecido e ajuda a repensar uma série de relacionamentos e interconexões dos sentimentos entre ser, estar e viver aqui, agora e para o futuro. Broom & Kenny afirmam que se voltar a esse *corpus* é entender que há um ator produtivo em cena e que deve estar visível nos sistemas das relações afetivas com familiares, cuidadores, profissionais e estruturas institucionais de tratamento e cuidado.

O capítulo 2, *Waiting, Hauntings and Surviving*, foi escrito a partir dos diários que as pessoas com câncer foram convidadas a produzir e discute a “espera no câncer”. “*O que se espera, por quem esperar, com que fim se espera*” (p. 49-50), “...*quando a espera termina?*” (p. 59). As relações e as expectativas produzem a espera como prática social, que altera a linearidade do tempo e cria “assombrações” que colocam a doença em primeiro plano - o poder disciplinar e normativo - e a pessoa adoecida como plano de fundo - vivendo sob um prognóstico e tolerando os efeitos das intervenções tensionadas pela vigilância, atribuídas sob moralidades normativas e pesadas responsabilidades.

O capítulo 3, *Malignant Attitudes*, aborda a “atitude” das pessoas no contexto da vida com o câncer como uma entidade complexa, “*implantada culturalmente*” (p. 65), em conformidade com o domínio moral e ético. Os entrevistados apontam criticamente que a “*atitude tem vida própria no contexto do câncer*” (p. 66) e manter a “*boa atitude*” remete a moralidades e ideias dominantes relacionadas ao “*bom paciente*” (p. 77), à “*cidadania produtiva*” (p. 66) e à excelência do comportamento desejável, com a firmeza e coerência do agir e reagir.

O capítulo 4, *Entangled and Estrange*, discute a sociabilidade do entrelaçamento e da inclusão nas relações afetivas daqueles que vivem com o câncer e sua rede de apoio. Os entrevistados destacaram, na sua vida cotidiana em *survivorship* (sobrevivência), as relações de des/continuidade, o evento de “estar aqui” e “ir embora”, a presença do “eu” e do “outro” e a ruminação de “*rever o passado, viver o momento e garantir o futuro*” (p. 96). Destacaram, ainda, a experiência de “*exilados do passado, suspensos do presente e excluídos do futuro*” (p. 96) estabelecendo emaranhados de corpos, de “eus”, de coisas, do tempo e das relações no espectro de uma “*fenomenologia do viver com o câncer*” (p. 94).

O capítulo 5, *Collective Emotions, Affective Relation*s, mostra que o câncer não é um projeto individual e sim coletivo, ou “*multiperspectivo das relações entre corpos, sujeitos, discursos e práticas*” com “*crenças, desejos e normas*” (p. 100). É uma “economia afetiva e moral” de controle, moderação ou dominação. Por um lado, há uma dinâmica de obrigação das pessoas se manterem perseverantes e otimistas mesmo estando em estágio de “*medo, pavor, desesperança e melancolia*” (p. 107) diante do câncer. Por outro lado, pergunta-se: ser obediente resulta numa vida boa ou numa vida apenas mais longa?

O capítulo 6, *Enchantment, Acceleration and Innovation*, debate a relação entre a medicina de precisão na sobrevida de pessoas com alguns tipos de câncer e os aspectos econômicos, culturais, e profissionais no processo de *survivorship*. Muitas questões ficam sinalizadas e não respondidas, entre elas: o projeto de precisão valoriza o tempo de vida ou a extensão da vida sobre a finitude? Ou ainda: a cura em oncologia é um desejo humano ou uma imposição da indústria de inovação da saúde?

O capítulo 7, *Participation and the Making of Possibility*, complementa o capítulo anterior acerca da “*ascensão da medicina de precisão em oncologia, a aceleração dos ensaios clínicos oferecidos*” (p. 137) e a mercantilização ideológica e econômica. A obstinação terapêutica para manter a vida e a sobrevivência ao câncer curvam-se ao mercado e trazem novas questões sobre “*valor, custo, investimentos e interesses, com distanciamento da assistência social e bem-estar*” (p. 137).

O livro apoia-se em referenciais teóricos e ontológicos do câncer (fenomenologia da espera; indústria das atitudes morais; sociologia das emoções; entrelaçamentos da temporalidade; conectividades e estranhamentos; tecnologia das inovações e precisão; e ensaios clínicos) que ampliam as reflexões acerca da “socio-lógica” do câncer e das relações de poder no processo de *survivorship*. Ao longo de 160 páginas, Alex Broom & Katherine Kenny descrevem o câncer como um fenômeno social coletivo permeado por diferentes formas de relacionalidade e normatividade. Valorizam as experiências narrativas dos participantes, produzidas com diferentes métodos de produção de informação (diários e fotografias produzidos pelos participantes, entrevistas abertas com diferentes atores e observações em serviços de saúde) e mapeiam uma “*sociologia crítica da sobrevivência*” (p. 6) que governa corpos, identidades e relações sociais. Os autores expandem a compreensão sobre a sobrevivência ao câncer, avançando os debates para além dos eventos de diagnóstico, prognóstico, tratamento, remissão e desfecho. Exploram as experiências daqueles que sobrevivem com o câncer e não daqueles que estão além dele, com “*uma voz sociológica mais forte*” (p. 10) para responder às questões silenciadas pela indústria, cultura de tratamentos, práticas terapêuticas e modelos institucionais que, na maioria das vezes, pouco valorizam a subjetividade no processo do sobreviver com o câncer.

Produzir uma “sociologia do câncer” exige contextualizar o evento nas diferentes esferas biológicas, sociais, culturais, políticas e econômicas, das quais emergem as questões relacionadas aos marcadores sociais das diferenças com as inúmeras formas de estigma, preconceito, discriminação, segregação, medo, coerção, exploração, vulnerabilidade, encantamento, aceleração, inovação, precisão, prazeres e outros. Portanto, exige uma complexidade multidimensional que deve ser discutida, também, à luz do paradigma da interseccionalidade ^2^, da politicidade do cuidado ^3^ e do cuidado emancipador ^4^.

O livro *Survivorship: A Sociology of Cancer in Everyday Life* é um extenso exercício sociológico que deve ser explorado por pesquisadores, estudantes e profissionais da saúde interessados não apenas em compreender a construção social das relações entorno de uma doença, mas também os sentidos, significados e representações culturais que povoam as mentes e os corações daqueles que cuidam e que são cuidados.
